# Editorial

**Published:** 1875-12

**Authors:** 


### Editorial.

### THE LIST OF DENTISTS IN OHIO.

We have just received the list of Dentists of Ohio, published by direction of the Ohio State Society, from it we learn that there are, in the state, one hundred and three graduates, and one hundred and forty-two who have passed the Board of Examiners, and have a certificate of qualification, and two hundred and ninety-six who have neither; five hundred and forty-one in all. Of the latter class doubtless by far the larger proportion are practicing in accordance with the law as amended, the purport of which was, that all dentists who had been in practice over five years, previous to the passage of the law, were regarded as having complied with its requirements. There are very few practicing in the state in violation of the law. A few there may be, who are going along and striving to make preparation for an examination.

This list will be a matter of interest to every dentist in the state, by it some estimation may be made of the legal status at least, of each dentist in the state, not as fully however, as it might have been, by filling the blanks in the column indicating the licenses and graduates, with the number of years of practice, of each man respectively. This would have shown at once how many are practicing in violation of the law. We notice quite a number of mistakes in the spelling of names, the initials, etc.

We will say, in the way of apology for the Secretary of the Society, who supervised this work, that he was placed in in rather peculiar and embarassing circumstances, had his attention somewhat diverted during the time this work was being done. This list will require revision within a year or two, and then all corrections and emendations can be made.

### THE DENTAL LAW IN OHIO.

The question has sometimes been asked, has the law regulating the practice of dentistry, accomplished any good? and what will be its influence in the future?

In answer to this, it may be said, that far greater results have been attained thus far than the most sanguine hoped for. Its influence has caused a large number, especially of the younger members of the profession in the state, to enter upon a course of study and preparation, in order to answer the requirements of the law, and to better prepare themselves for practice; incalculable benefit has been derived in this respect. Most of such persons have presented themselves for examination as to qualification. This has exercised quite a stimulating effect upon even the older members of the profession. Another very palpable advantage obtained, is the arrest of the preparation and entrance to the practice of the profession of incompetent and unworthy men. No young man now finds it practicable or even possible, if proper attention is given to the subject, to enter an office for a pupilage of a few weeks or a few months and then start into practice upon his own responsibility. Nearly all who enter the practice now in the state, have graduated from some respectable college.

In this fact we have great promise for the future. Thus far the law has accomplished more than was expected.

Its efficiency in the future will depend very much upon the attention given to it by the profession throughout the state. It is a matter of interest to every dentist, that the law should be sustained and that it should not be permitted to fall into disuse. The law is just and right, aud a full compliance with its requirements, will be for the benefit of all.

Some have questioned its efficiency, because a large number of prosecutions have not been instituted under it. In reply to this criticism, it may be said, it has not been necessary because the acquiesence with its provisions has been so nearly universal. This instead of being an argument against the law, is one of the strongest for it.

### CARE OF THE TEETH DURING GESTATION.

This is a subject of very great importance, notwithstanding very little direct attention has been given to it. That the teeth always suffer more or less, and in many instances very greatly, during these periods, none who have given much attention to the subject will deny.

Teeth of good structure, and that have maintained their integrity perfectly up to the period of gestation, very soon afterward begin to give indications of frailty. First by susceptibility to irritation under influences that previously would have made no impression. A sense of uneasiness is first experienced, more or less pronounced, after a short time actual pain ensues. Very frequently during this period, a change in the density of the teeth take place, that is quite apparent to the close observer. So marked are the injuries which the teeth sustain during child bearing and rearing, that a proverb has obtained amongst the common people to this effect, viz: "That for every child a tooth is lost."

There are doubtless very great differences of susceptibility in different cases in this respect, yet almost all suffer more or less, and recognize the fact too, and would be rejoiced to know that relief was possible.

The question occurs here, why this general and marked susceptibility to irritation? An answer to this question may be found, in the fact that the nervous system during gestation is brought under a tension and a stress of function, never before experienced, nor after relief from the condition is obtained.

And again the life force is so diverted and drawn to another point of action, that the teeth (and this perhaps is true of many other organs,) are not possessed of the full tide and tone of vital endownment as before, and so are less resistant to irritating and disease producing influences.

At such periods the nutrition of the teeth is far less perfect, owing to weakened sustaining power, together with, in a great many cases at least, a want of the proper pabulum for their support. This deficiency of nutrient material occurring either because it is not received in the food or if received, is not assimilated and appropriated in sufficient quantity, and in a manner to satisfy, the great and increased demand for it.

A very large part of the nutrient material taken into the blood of the mother is required and appropriated for the growth and development of the new being. It is very probable that the original design in this process, was that the assimilating and appropriating function, should be so enlarged in its action, as to provide pabulum to answer the double demand of complete support to the formed organs of the mother, and supply to the growing fœtus. But so much interference has been made with nature's plans, and so great interruption of her processes and functions, that full conformity thereto is no where found; as a result of this, the conditions we have referred to are found.

And now what shall be done to meet the requirements. We may recognize the fact here at once that a complete return to nature's original plan can not be effected in one, two or three generations. It will take many, even though all ive up to the fullest light on the subject. Any course of treatment of the mother during gestation, that will subserve the welfare of her teeth, will tend in the same direction, for all the organs and tissues of the body.

Mere local treatment of the teeth and the parts about them will amount to little or nothing so far as changing the condition and susceptibility is concerned. It is true that then as at all other times, the teeth should be kept scrupulously clean and the mouth in a healthy condition, and when the mouth has been previously in a bad condition, local palliative treatment may be required and serve the purpose of mitigating pain and suffering. But in all cases the treatment to meet such emergencies should be more than a mere local matter. The real demand is through the system; and the first of all, the toning up and invigorating the life forces, strengthening functional action. Build up the sustaining power. The mother should receive in her food, all the constituents required to form the pabulum for each and every tissue of the body, and especially for the teeth, as it is to them we are now directing attention.

Her digestion should be especially guarded in every respect and kept as nearly to its maximum working ability,as is compatible with entire safety. Let the assimilating and appropriating functions be invigorated and strengthened, as far as possible.

Now it is not only necessary, that the supporting power should be maintained and that the nutrient function in every step, should receive attention, guidance and support, but the excretory functions of the body must receive attention as well.

In order that new material may be properly appropriated, either for sustaining fully developed tissue or for supplying growing organs, it is necessary that the debris and waste material be promptly removed. All the excretory organs should be maintained in the best possible condition, the functions of each and every one should be well performed. The skin, the kidneys, the liver and the lungs should each perform its work with promptness and energy, the greatest immunity should be secured from malarial and all subtle, poisonous or pernicious influences, excluding from the body, every and all things that would operate adversely to its most perfect well being.

Now in these suggestions, we haye only aimed to outline that which every well informed dentist should be able to fill up and completely round out for himself.

### THE CLOSE OF THE VOLUME.

With this number closes the twenty-ninth volume of the Register, what it has been and what it has accomplished, its readers already know; and the good done, is in proportion to the number of its readers, and the faithful support which they with others have given it.

It is a matter of no small moment and responsibility, to meet from fifteen hundred to two thousand members of our profession every month, face to face, on matters pertaining to every day practice, that influence, more or less' the welfare and comfort of a great many thousands of people.

How often is it, that the dentist is misled, by false principles or practice, or by the vague and indefinite expression of correct principles or practices.

We have received very often, suggestions and directions in reference to the management and conducting of the Register. We are thankful and glad for any thing calculated to make the Register better.

What the Register will be in future, will depend perhaps quite as much upon the contributors to its pages, and upon the support it receives in other respects, as upon its editor We trust that during the coming year, the Register will wield a wider and deeper influence than in the past. We hope to secure some special aid during the coming year, which we know will be highly appreciated by the profession.

---

MARRIED.—Oct. 21st, 1875, Dr. J. M. Porter, of Toledo, O. to Miss Mary Folger, of Massillon, O., at the brides' home. This announcement will be received with pleasure by Dr. P's. friends, and especially so, since the impression was entertained in some quarters, that he was rather opposed to such proceedings, and was inclined to fight it out on another line.

May the joys of the happy pair ever flow on, and their happiness brighten throughout life's journey.

